# Muscle strength rather than appendicular skeletal muscle mass might affect spinal sagittal alignment, low back pain, and health-related quality of life

**DOI:** 10.1038/s41598-023-37125-w

**Published:** 2023-06-19

**Authors:** Yoshihide Tanaka, Masayuki Miyagi, Gen Inoue, Yusuke Hori, Kazuhide Inage, Kosuke Murata, Hisako Fujimaki, Akiyoshi Kuroda, Yuji Yokozeki, Sho Inoue, Yusuke Mimura, Shinji Takahashi, Shoichiro Ohyama, Hidetomi Terai, Masatoshi Hoshino, Akinobu Suzuki, Tadao Tsujio, Hiromitsu Toyoda, Sumihisa Orita, Yawara Eguchi, Yasuhiro Shiga, Takeo Furuya, Satoshi Maki, Shinsuke Ikeda, Eiki Shirasawa, Takayuki Imura, Toshiyuki Nakazawa, Kentaro Uchida, Seiji Ohtori, Hiroaki Nakamura, Masashi Takaso

**Affiliations:** 1 Department of Orthopaedic Surgery, School of Medicine, Kitasato University, 1-15-1 Kitasato, Minami-Ku, Sagamihara, Kanagawa 252-0375 Japan; 2 Department of Orthopaedic Surgery, Osaka Metropolitan University Graduate School of Medicine, Osaka, Japan; 3 Department of Orthopaedic Surgery, Graduate School of Medicine, Chiba University, Chiba, Japan; 4 Department of Orthopaedic Surgery, Osaka City General Hospital, Osaka, Japan; 5 Department of Orthopaedic Surgery, Shiraniwa Hospital, Nara, Japan; 6 Center for Frontier Medical Engineering, Chiba University, Chiba, Japan

**Keywords:** Epidemiology, Neurological disorders

## Abstract

Sarcopenia is defined as decreasing in muscle strength and mass, and dynapenia is defined as decreasing in muscle strength and maintained muscle mass. This study elucidated the prevalence and characteristics of sarcopenia and dynapenia and evaluate in elderly spinal disorders patients. 1039 spinal disorders patients aged ≥ 65 years were included. We measured age, grip strength, muscle mass, spinal sagittal alignment parameters, low back pain (LBP) scores and health-related quality of life (HR-QoL) scores. Based on the previous reports, patients were categorised into normal group: NG, pre-sarcopenia group: PG, dynapenia group: DG, and sarcopenia group: SG. Pre-sarcopenia, dynapenia, and sarcopenia were found in 101 (9.7%), 249 (19.2%), and 91 (8.8%) patients, respectively. The spinal sagittal alignment parameters, trunk muscle mass, LBP, and HR-QoL scores were significantly worse in DG and SG compared with those in PG and NG. Spinal alignment, trunk muscle mass, and clinical outcomes, including LBP and HR-QoL scores, were maintained in the PG and poor in the DG and SG. Thus, intervention for muscle strength may be a treatment option for changes of spinal sagittal alignment and low back pain.

## Introduction

Decreasing muscle strength and muscle mass have recently received increasing attention. Rosenberg et al. first reported sarcopenia, defined as loss of muscle strength and muscle mass due to aging^1^. It has been reported sarcopenia induced to a high risk of mortality and incident disability^2^. Additionally, sarcopenia showed a significant financial burden, with medical expense for sarcopenia in the US in 2000 amounting to approximately $18.5 billion^3^. In contrast, dynapenia, a condition in which maintained muscle mass but reduced muscle strength, has also attracted attention. It has also been reported that dynapenia was highly associated with mortality and physical disfuction^4^, similar to sarcopenia. Moreover, it has been reported that muscle quality and strength, rather than muscle mass, are important clinical findings including mortality risk and physical performance^5,6^. Therefore, we should pay attention to muscle strength as well as muscle mass when treating patients with musculoskeletal disorders, such as those with spinal diseases.

Regarding the muscle in patients with spinal diseases, the prevalence of sarcopenia was higher in patients with spinal canal stenosis compared to normal subjects^7^. In addition, the prevalence of sarcopenia was higher in patients with spinal deformity than in those with spinal stenosis^8^. Regarding clinical findings, patients with spinal diseases with sarcopenia showed poor LBP scores and outcomes after surgery^9,10^. However, no reports have evaluated spinal alignment or clinical findings in spinal diseases with dynapenia. Determining whether muscle mass or muscle strength is more important would be helpful when treating patients with spinal diseases; however, this remains unclear. In particular, only a few reports focused on the differences between muscle mass and muscle strength as factors affecting clinical findings including spinal alignment and low back pain in elderly. Therefore, we hypothesized that decreased muscle mass or muscle strength affects clinical findings, spinal alignment, and body composition.

For testing this hypothesis, the purpose of the current study were to elucidate the prevalence of sarcopenia and dynapenia and evaluate whether muscle mass or muscle strength affect clinical outcomes such as low back pain (LBP) and spinal sagittal alignment in elderly spinal disorders patients.

## Methods

We obtained ethical approval from our institutional review board for the current study. Further, we also conducted the current study according to the 1964 Declaration of Helsinki and its later amendments.

### Patients and measurement items

This cross-sectional and observation study was a multicentre involving nine facilities. We included spinal diseases patients aged ≥ 65 years, whose diagnosis were vertebral fractures, spinal stenosis, spinal deformities, spinal tumours, and osteoporosis, in the current study. Because a myelopathy may affect grip strength, we excluded patients with myelopathies such as cervical spondylosis and cervical disc herniation. We reviewed the age, gender, and anamnestic medical past history from the medical charts of all cases. Body composition, grip strength, lateral X-rays of the whole spine including hip joints on the standing position, and clinical findings, including LBP and health-related quality of life (HRQoL) scores, were also evaluated.

### Body composition measurements

We assessed several body composition parameters using bioelectrical impedance analysis (BIA) methods. MC-780A or MC-980A body composition analyser (Tanita Co., Tokyo, Japan) was used in all cases. Patients with a pacemaker were excluded because the body composition could not be evaluated. Moreover, we excluded data from patients with implant and instrumentation in their body because of their measurement reliability. Next, we calculated several following parameters.

Skeletal muscle mass index (SMI: the corrected appendicular skeletal muscle mass) = appendicular skeletal muscle mass (kg)/(body height(m))^2^ (kg/m^2^).

Trunk muscle mass index (TMI: the corrected trunk muscle mass) = Trunk muscle mass (kg)/(body height(m))^2^ (kg/m^2^).

### Spinal sagittal alignment and clinical outcome evaluation

**Figure 1:**
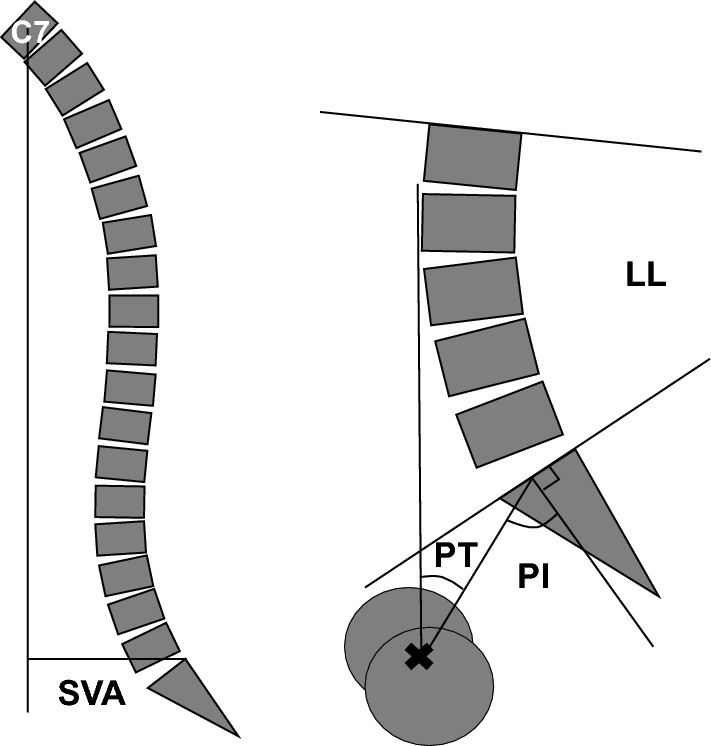
The spinal sagittal alignment measurements. *C7* cervical 7, *SVA* sagittal vertical axis, *PT* pelvic tilt, *PI-LL* pelvic incidence minus lumbar lordosis.

To evaluate spinal sagittal alignment, we evaluated three parameters, including the pelvic tilt (PT), sagittal vertical axis (SVA), and pelvic incidence minus lumbar lordosis (PI − LL), on lateral X-rays of the whole spine including hip joints on the standing position, as previously reported^11,12^. (Fig. 1) We evaluated LBP scores using the visual analogue scale (VAS) of LBP in the last week and the Oswestry Disability Index (ODI) score. In addition, for evaluating HRQoL, we used the EuroQol 5 Dimension (EQ5D).

### The definition of pre-sarcopenia, dynapenia, and sarcopenia

**Figure 2:**
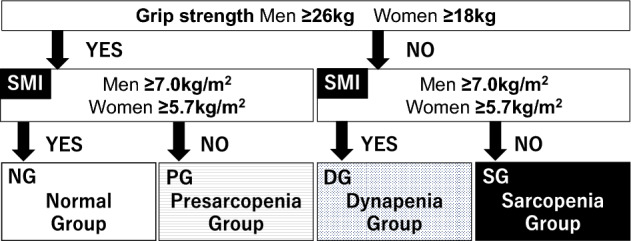
The definitions of pre-sarcopenia, dynapenia, and sarcopenia. *SMI* Skeletal muscle mass index.

As previously reported^5^, we divided patients into four groups: a normal group (NG), comprising normal muscle strength and normal appendicular skeletal muscle mass; a pre-sarcopenia group (PG), comprising normal muscle strength and decreased appendicular skeletal muscle mass; a dynapenia group (DG), comprising decreased muscle strength and normal appendicular skeletal muscle mass; and a sarcopenia group (SG), comprising decreased muscle strength and decreased appendicular skeletal muscle mass. Based on the classification of the Asian Working Group for Sarcopenia^13^, decreased muscle strength was defined that grip strength was less than 26 kg for men and less than 18 kg for women. Moreover, decreased muscle mass was defined that SMI were less than 7.0 kg/m^2^ for men and less than 5.7 kg/m^2^ for women. (Fig. 2). The prevalence of pre-sarcopenia, dynapenia, and sarcopenia was calculated.

### Statistical analysis

As statistical analysis, we performed one-way analysis of variance (ANOVA) for comparing the mean of all measurements among the four groups. Then, we performed Post hoc analysis using Tukey’s test for multiple comparisons. We performed these comparisons separately for men and women, since spinal alignment parameters, clinical findings, body composition parameters, and grip strength may differ between the two sexes. We also used Pearson’s correlation coefficients to evaluate the relationships between muscle mass or strength and spinal alignment or clinical outcome. Because muscle mass and strength differ between men and women, we performed these comparisons separately for both sexes. The correlation coefficients were delineated: R-values of 0.2–0.4, 0.4–0.7, and 0.7–1 were considered weak, moderate, and strong correlations, respectively. We analysed all data using IBM SPSS Statistics version 26 (IBM, Armonk, NY, USA), and considered significant which a p-value was less than 0.05.

### Ethics approval

Ethical approval from the Institutional Review Board of Kitasato University was obtained for this study, which was conducted in accordance with the ethical principles specified in the 1964 Declaration of Helsinki and its later amendments. The approval code is B18-086.

### Informed consent statement

Informed consent was obtained from all subjects involved in the study.

## Results

### The prevalence of pre-sarcopenia, dynapenia, and sarcopenia

In total, 1039 patients (mean age, 74.6 years; 445 men, 594 women) were analysed in the current study. Among these, 648 (62.4%), 101 (9.7%), 199 (19.2%), and 91 (8.8%) patients were classified into the normal, pre-sarcopenia, dynapenia, and sarcopenia groups, respectively. For men, 326 (73.3%), 46 (10.3%), 52 (11.7%), and 21 (4.7%) patients were classified into the normal, pre-sarcopenia, dynapenia, and sarcopenia groups, respectively. For women, 322 (54.2%), 55 (9.3%), 147 (24.7%), and 70 (11.8%) patients were classified into the normal, pre-sarcopenia, dynapenia, and sarcopenia groups, respectively.

### Multiple comparison analysis

**Figure 3:**
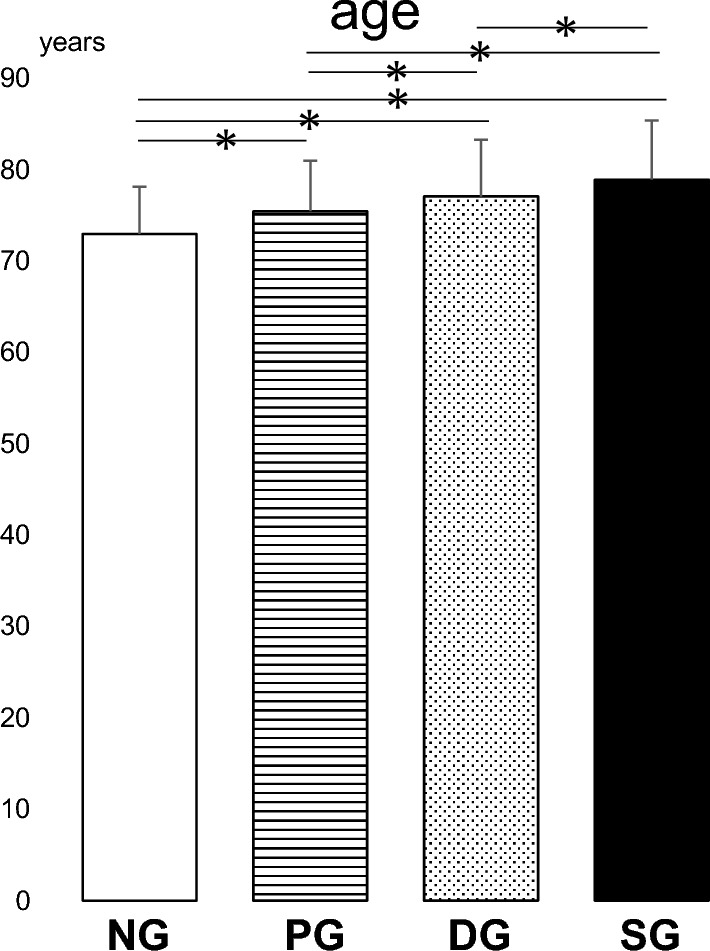
The results of the multiple comparison analysis for age among the four groups (*NG* the normal group, *PG* pre-sarcopenia group, *DG* dynapenia group, *SG* sarcopenia group) are shown. *p < 0.05.

Mean age gradually increased in the following order: NG, PG, DG, and SG. The mean ages in SG, DG, and PG were significantly higher than that in NG (p < 0.05). Mean ages in SG and DG were also significantly higher than that in PG (p < 0.05). Furthermore, statistically significantly higher mean age was observed in SG compared with DG. (p < 0.05) (Fig. 3).

**Figure 4:**
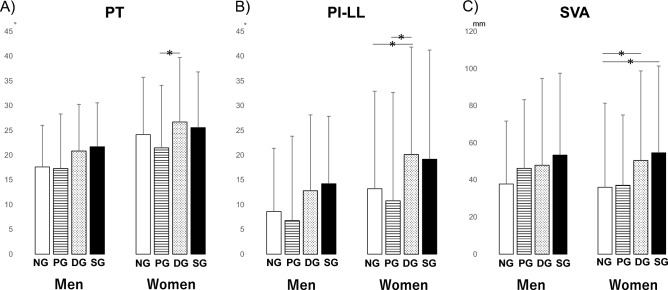
The results of the multiple comparison analysis for spinal sagittal alignment parameters among the four groups (*NG* the normal group, *PG* pre-sarcopenia group, *DG* dynapenia group, *SG* sarcopenia group) are shown. (**A**) *PT* pelvic tilt (**B**) *PI − LL* pelvic incidence minus lumbar lordosis (**C**) *SVA* sagittal vertical axis. *p < 0.05.

Regarding the spinal sagittal plane alignment, we analyzed the data on men and women separately. Female patients in the DG had a significantly higher PT than those in the NG (p < 0.05) (Fig. 4A). Similarly, significantly higher PI − LL was observed in women in the DG than in the NG (p < 0.05) (Fig. 4B). In addition, female patients in the DG and SG had a significantly higher SVA than those in the NG (p < 0.05) (Fig. 4C).

**Figure 5:**
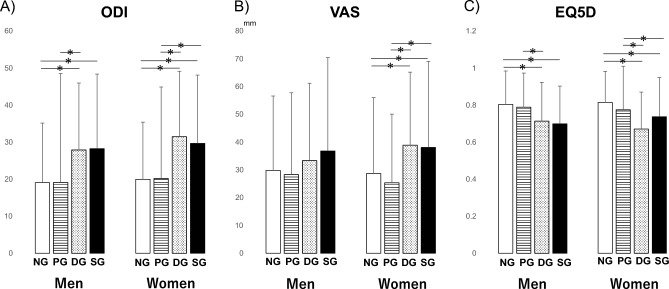
The results of the multiple comparison analysis for clinical outcomes among the four groups (*NG* the normal group, *PG* pre-sarcopenia group, *DG* dynapenia group, *SG* sarcopenia group) are shown. (**A**) *ODI* oswestry disability index (**B**) *VAS* visual analogue scale of low back pain (**C**) *EQ5D* EuroQol 5 dimension. *p < 0.05.

For the clinical findings, similar trends were observed for the ODI, VAS, and EQ5D scores in men and women. For men, the ODI scores in the DG and SG were significantly higher than those in the NG (p < 0.05). In addition, the ODI scores in the DG were significantly higher than those in the PG (p < 0.05). For women, the ODI scores in the DG and SG were significantly higher than those in the NG and PG (p < 0.05) (Fig. 5A). In addition, the VAS scores for LBP in the DG and SG were significantly higher than those in the NG and PG (p < 0.05) (Fig. 5B). For men, the EQ5D scores as HR-QoL scores in the DG and SG were significantly higher than those in the NG (p < 0.05). In addition, the EQ5D scores in the DG were significantly higher than those in the PG (p < 0.05). For women, the EQ5D scores in the DG and SG were significantly higher than those in the NG and PG (p < 0.05) (Fig. 5C).

**Figure 6:**
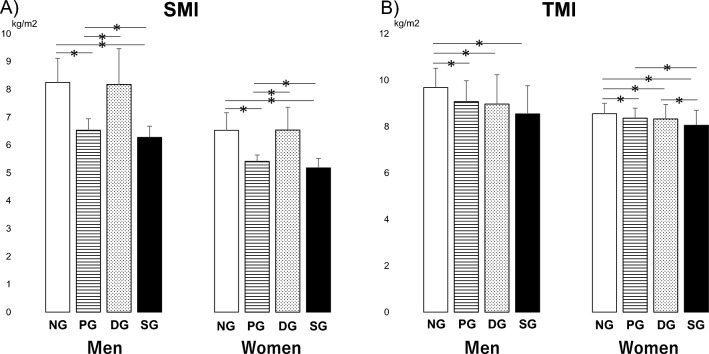
The results of the multiple comparison analysis for muscle mass index among the four groups (*NG* the normal group, *PG* pre-sarcopenia group, *DG* dynapenia group, *SG* sarcopenia group) are shown. (**A**) *SMI* Skeletal muscle mass index (**B**) *TMI* Trunk muscle mass index. *p < 0.05.

For body composition, we analysed men and women separately. Regarding muscle mass, in both men and women, patients in PG and SG had significantly lower SMI than patients in DG and NG (p < 0.05) (Fig. 6A). However, interestingly, a different tendency was observed for the TMI than for the SMI. In men, patients in PG, DG, and SG had significantly lower TMI compared with patients in NG (p < 0.05). In contrast, no significant difference in the TMI was observed between PG and DG (p > 0.05). In women, patients in PG, DG, and SG had significantly lower TMI compared with patients in NG (p < 0.05). Moreover, the TMI in SG was significantly lower than that in PG and DG (p < 0.05). In contrast, no significant difference in the TMI was observed between PG and DG (p > 0.05) (Fig. 6B).

**Figure 7:**
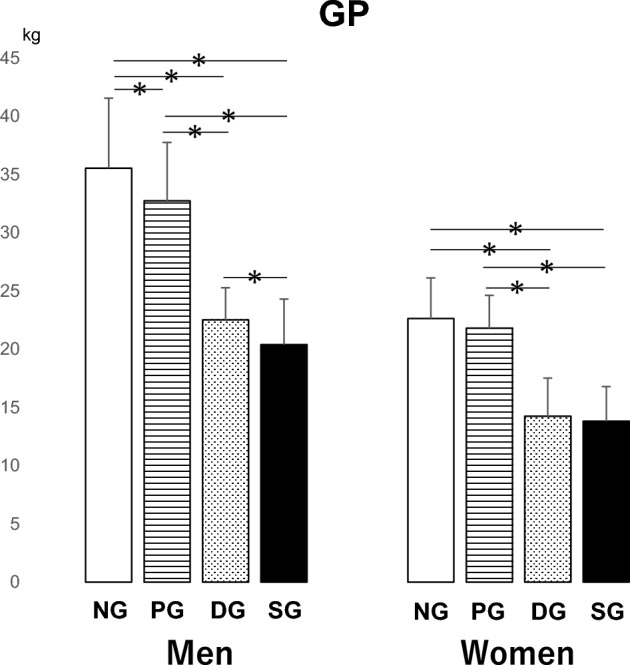
The results of the multiple comparison analysis for grip strength among the four groups (*N* normal, *P* pre-sarcopenia, *D* dynapenia, and *S* sarcopenia groups) are shown. *p < 0.05.

For men, the mean grip strength gradually decreased in the following order: NG, PG, DG, and SG. The mean grip strengths in the SG, DG, and PG were significantly lower than those in the NG (p < 0.05). The mean grip strengths in the SG and DG were also significantly lower than those in the PG (p < 0.05). Furthermore, a statistically significantly lower mean grip strength was observed in the SG than in the DG (p < 0.05). For women, the mean grip strengths in the DG and SG were significantly lower than those in the NG and PG (p < 0.05) (Fig. 7).

Table 1 shows the correlations between muscle mass or strength and spinal alignment or clinical outcome. For men, grip strength significantly negatively weakly correlated with PI-LL, SVA, and ODI (r =  − 0.208, p = 0.000; r =  − 0.217, p = 0.000; and r =  − 0.252, p = 0.000, respectively). In addition, a significant positive weak correlation was identified between grip strength and EQ5D (r = 0.227, p = 0.000). For women, grip strength also significantly negatively weakly correlated with PI-LL, SVA, and ODI (r =  − 0.239, p = 0.000; r =  − 0.224, p = 0.000; and r =  − 0.358, p = 0.000, respectively). In addition, a significant positive weak correlation was identified between grip strength and EQ5D (r = 0.227, p = 0.000). A significant positive weak correlation was also found between grip strength and EQ5D (r = 0.351, p = 0.000). In contrast, SMI was not significantly correlated with all spinal sagittal alignment parameters and clinical outcomes for men and women.

**Table 1:** The correlations between SMI or grip strength and spinal alignment or clinical outcome.

		PT	PI-LL	SVA	ODI	VAS	EQ5D
Men							
SMI	*r*	0.017	0.072	− 0.013	− 0.007	0.064	0.022
	*P*	0.734	0.145	0.789	0.877	0.194	0.651
Grip strength	*r*	− 0.188	** − 0.208**	** − 0.217**	** − 0.252**	− 0.081	**0.227**
	*p*	0.000	**0.000**	**0.000**	**0.000**	0.098	**0.000**
Women							
SMI	*r*	0.065	0.006	0.031	0.007	0.017	− 0.018
	*p*	0.128	0.893	0.477	0.862	0.690	0.684
Grip strength	*r*	− 0.189	** − 0.239**	** − 0.224**	** − 0.358**	− 0.182	**0.351**
	*p*	0.000	**0.000**	**0.000**	**0.000**	0.000	**0.000**

Significant values are in bold.

## Discussion

In this study, the incidence rates of pre-sarcopenia, dynapenia, and sarcopenia were 9.7%, 19.2%, and 8.8%, respectively, indicating that 37.6% of elderly patients with spinal diseases showed decreased muscle mass or muscle strength. Additionally, the age in PG, DG and SG was significantly higher compared with that in NG. Several authors have reported that decreased muscle strength or mass is highly related with ageing in elderly patients^14,15^. Additionally, muscle strength decline is more rapid than muscle mass decline^14^. Petermann-Rocha et al. reported in their systematic review that the incidence of sarcopenia varied between 10 and 27% because subjects, cut-off points, and classifications were different among these studies^16^. These findings indicated that there were unexpectedly many patients with spinal diseases with decreased muscle mass or strength. In addition, ageing may be a risk factor for loss of muscle strength and mass.

Regarding spinal alignment, in the current study, the groups with decreased muscle strength, not the pre-sarcopenia group showing decreased muscle mass, showed changes of spinal sagittal alignment compared to the normal. In addition, several spinal sagittal alignment parameters, including PI-LL and SVA, significantly correlated with grip strength but not with SMI. Several authors have reported that decreased appendicular skeletal muscle mass was related with spinal sagittal malalignment in spinal diseases patients^17–19^. In addition, a previous report showed that low trunk muscle mass and grip strength might be risk factors for spinal malalignment in spinal diseases patients^20^. Thus, muscle strength as well as muscle mass were associated with spinal sagittal alignment. However, there were few reports to elucidate which of muscle mass or strength was strongly associated with spinal alignment. Kudo et al. reported that back extensor muscle strength and quality, but not appendicular skeletal muscle mass, was related with spinal sagittal alignment in elderly patients^21^. Based on these findings from the current study and previous reports, muscle strength, rather than appendicular skeletal muscle mass, might be more strongly associated with spinal sagittal alignment.

For the LBP scores and HRQoL score, similar to results from spinal alignment, the groups that showed decreased muscle strength, not the pre-sarcopenia group, had deteriorated LBP score including ODI ans VAS of LBP and EQ5D score than the normal. The ODI and EQ5D also significantly correlated with grip strength but not SMI. Spinal alignment may be strongly associated with clinical findings. Matsuyama stated in his review that patients with spinal sagittal malalignment usually have various disabilities due to LBP^22^. In addition, several spinal sagittal alignment parameters have been reported to be strongly associated with HRQoL scores^23–25^. In another previous report, patients with osteoporosis and spinal sagittal malalignment showed deteriorated LBP scores^26^. Moreover, it has been reported that improving spinal sagittal alignment after corrective surgery improved the HRQoL scores^27^. These findings from the previous reports indicated decreasing muscle strength as well as muscle mass were associated with changes of spinal sagittal alignment leading to low back pain. However, there were no reports to demonstrate which of muscle strength or muscle mass would be important for low back pain. Cobmined with findings from the present study, it was suggested that decreasing muscle strength, but not loss of muscle mass, might cause the deterioration of clinical findings, including LBP and HRQoL, via the deterioration of spinal alignment.

In this study, patients in the dynapenia group with decreased muscle strength had significantly lower trunk muscle mass than patients in the normal group, although the appendicular skeletal muscle mass was preserved. Thus, trunk muscle mass, rather than appendicular skeletal muscle mass, may be important, especially in spinal diseases patients. Yamamoto et al. reported that a low trunk muscle mass was related with hyperkyphosis but not appendicular skeletal muscle mass^28^. A previous report indicated the trunk muscle mass was significantly related with spinal alignment, ODI scores, and EQ5D scores when adjusted for appendicular skeletal muscle mass^29^. Furthermore, a decreased trunk muscle mass might be a possible risk factor for LBP in osteoporosis patients^30^. Thus, trunk muscles might be an important factor for maintaining posture and improving clinical findings and should be considered when treating patients with spinal diseases.

The finding of the current study that muscle strength and trunk muscle mass, rather than appendicular skeletal muscle mass, might affect spinal sagittal alignment, LBP, and HRQoL is of clinical relevance. Several authors reported that even if muscle mass was lost, mortality was not increased if the muscle strength was maintained^31,32^. In addition, it has been reported exercise therapy had a positive effect on muscle strength, but there is insufficient evidence for muscle mass^33^. Therefore, exercise therapy for muscle strength may be important for treating patients with spinal diseases.

There were some limitations in the current study. First, this study was a cross-sectional study, and it was unclear whether muscle strength affects spinal alignment and clinical findings or whether spinal alignment and clinical findings affect muscle strength. A longitudinal study is needed in the future to evaluate these aspects. Second, other factors, such as vertebral fractures, may be related with body composition and spinal sagittal alignment. Nevertheless, we did not evaluate vertebral fractures in this study. Thus, some measurements, including the BMI, SMI, and TMI, might be overestimated in patients with vertebral fractures because body height might be underestimated in these patients. However, the method to correct this body composition remains unclear. Therefore, muscle mass can be compared without body height correction, and further longitudinal studies should be conducted in the future. Third, we were concerned about the accuracy of the measurement of trunk muscle mass using BIA because it included visceral and cardiac muscles. However, spinal alignment parameters and low back pain scores have been associated with trunk muscle mass, which was measured by BIA, but not skeletal muscle mass in patients with spinal diseases^29^. Therefore, the measurement of trunk muscle mass using BIA is worth evaluating, especially in studies of patients with spinal diseases. Fourth, we did not the measure trunk muscle strength. A discrepancy may exist between the tendency of grip strength and trunk muscle strength. Measuring the trunk muscle strength as well as grip strength may be important for evaluating spinal sagittal alignment and LBP, especially in patients with spinal diseases. Further studies are needed.

In conclusion, the current study showed that approximately 20% and 10% of the patients with spinal disorders had dynapenia and sarcopenia, respectively. In addition, patients with low muscle strength had low trunk muscle mass, poor outcomes of LBP, poor HRQoL, changes of spinal sagittal alignment, but elderly patients with low appendicular skeletal muscle mass alone did not. Thus, interventions for muscle strength may be a treatment option for changes of spinal sagittal alignment and LBP.

## Data Availability

The data are available on reasonable request to the corresponding author.
